# Memes in Medical Education

**DOI:** 10.1212/NE9.0000000000200281

**Published:** 2026-01-15

**Authors:** Stefano Sandrone

**Affiliations:** From the Department of Brain Sciences, Imperial College London, London, United Kingdom.

The term *meme*, coined by Richard Dawkins in 1976 in his book *The Selfish Gene*, originally described a “unit of culture” that is copied or imitated.^1^ With the rise of social media, memes have become a medium for expressing ideas and concepts concisely and in an entertaining way.^2^ Although the precise definition can vary across communities and over time,^3^ rapidity of dissemination and ironic touch are two key features.

Over the past two decades, memes have evolved into more complex structures, including text (catchphrases) and images, but also videos, songs or dances.^3^ Creating memes means “identifying a concept, choosing a still image or GIF that matches the concept, and writing captions”.^2^ Like many recent social media trends, their adoption was accelerated by the COVID-19 pandemic as they supplemented social interaction diminished by physical distancing.^4^ They are one of the most powerful and popular forms of communication, permeating both formal and informal education by blurring the boundaries between teaching, learning and “edutainment”.

Andragogically, meme creation can be mapped to the synthesis level in Bloom taxonomy, as learners create by summarizing.^2^ Some features of neurology might be well-suited to meme-ification. For example, many medical schools engage students in a curriculum that provides illustrations of the neural tracts, neuroanatomy and other figures to help them internalize complex concepts. The inherently visual nature of this material makes it especially well-suited for creative formats such as memes. Likewise, much of the neurologic examination is based on observation, an aspect that could easily be captured and conveyed through GIFs. Although research on memes in neurology and neuroscience education is still in its infancy, valuable insights into both meme creation and consumption can be drawn from other areas of medical education.

Medical students enrolled in a human physiology course were given the chance to create memes on renal physiology. Although only 52% of them (146 of 280) did so, thematic analysis revealed that memes elicited interest, facilitated peer interaction, simplified complex ideas, enhanced associated concept retention and fostered a positive learning environment.^4^ In another study, 200 third-year pharmacology students were asked to produce at least two work-appropriate memes in small groups.^5^ The outcomes were mixed, although mostly positive, as students viewed it as an opportunity to learn course content through creative methods while engaging in a “humanizing task” and connecting with fellow students. Difficulty understanding all the memes, likely due to a lack of contextual information, was reported.^5^

Learners' attitudes toward meme consumption are often positive, as memes positively impact the affective and cognitive domains, as shown by a cross-sectional survey with 119 second-year undergraduate students.^6^ They can increase learners' motivation and engagement^3^ and promote critical thinking.^7^ They can aid debriefing during a stressful period, as demonstrated in a study in which fifth-year medical students created and voted on memes related to course content and clinical rotations.^8^ Memes alluded to aspects that mirrored the students' state of mind and concerns, offering an opportunity to reflect on their humorous side.^8^ Ludic pedagogy may offer a complementary approach to teaching and interaction, formally and informally: within the boundaries of academic rigour, memes bring fun and playfulness, unleash creativity and facilitate student-led interaction.^8^

Using memes to reflect on professional identity formation has also been explored, and they induced a shift in the perception of twenty-six student pharmacists. Before submitting their memes, the students described their identity in an idealistic manner, portraying themselves as medication experts, educators and patient care providers. After seeing the memes submitted, their perception shifted to an identity primarily based on feelings of antagonism, being overwhelmed, undervalued and misperceived.^9^ Meme generation may serve as a medium for personal expression, fostering self-reflection and providing valuable insight into learners' experiences.

The risk of memes going wrong at different levels, from misinformation and violations of patient privacy to badmouthing hospitals or perpetuating cognitive and cultural stereotypes,^10^ is high and cannot be neglected. Students and staff can become cocreators, but some may be more reluctant to use memes when they are not fully confident in how to utilize creative digital methods.^3^ Educators, students, regulators and professional creators should work together to define standards and best practices in higher education^7^ and promote their ethical and responsible use. Research on professionalism, professional boundaries and what medical education memes should be (and not be) is urgently needed. Whether we are on the brink of a new era of “meme-dical education” remains to be seen, but dismissing memes *tout court* and ignoring their potential for both formal and informal learning would be shortsighted.
